# Targeted next-generation sequencing of a cancer transcriptome enhances detection of sequence variants and novel fusion transcripts

**DOI:** 10.1186/gb-2009-10-10-r115

**Published:** 2009-10-16

**Authors:** Joshua Z Levin, Michael F Berger, Xian Adiconis, Peter Rogov, Alexandre Melnikov, Timothy Fennell, Chad Nusbaum, Levi A Garraway, Andreas Gnirke

**Affiliations:** 1 Genome Sequencing and Analysis Program, Broad Institute of MIT and Harvard, 320 Charles Street, Cambridge, MA 02141, USA; 2 Cancer Program, Broad Institute of MIT and Harvard, 5 Cambridge Center, Cambridge, MA 02142, USA; 3 Sequencing Platform, Broad Institute of MIT and Harvard, 320 Charles Street, Cambridge, MA 02141, USA; 4 Department of Medical Oncology and Center for Cancer Genome Discovery, Dana-Farber Cancer Institute, Harvard Medical School, 44 Binney Street, Boston, MA 02115, USA

## Abstract

Targeted RNA-Seq combines next-generation sequencing with capture of sequences from a relevant subset of a transcriptome. When testing by capturing sequences from a tumor cDNA library by hybridization to oligonucleotide probes specific for 467 cancer-related genes, this method showed high selectivity, improved mutation detection enabling discovery of novel chimeric transcripts, and provided RNA expression data. Thus, targeted RNA-Seq produces an enhanced view of the molecular state of a set of "high interest" genes.

## Abstract 2

Combining next-generation sequencing with capture of sequences from a relevant subset of a transcriptome produces an enhanced view of this subset

## Background

In recent years, a technologic revolution has shifted DNA sequencing from traditional Sanger methods to "next-generation" sequencing (see review [1]). Applying these new sequencing methods to cDNA libraries, termed RNA-Seq, generates a wealth of information beyond that obtained from sequencing genomic DNA (see review [2]). RNA-Seq provides insights at multiple levels into the transcription of the genome as it yields sequence, splicing, and expression-level information leading to the identification of novel transcripts [3,4] and sequence alterations. For research into somatic mutations in cancer (for example, The Cancer Genome Atlas [5-7]), this method has the advantage of enriching for changes in coding sequences, which are more likely to affect function, compared with sequencing genomic DNA. Chromosomal rearrangements, including translocations, are an important class of mutations in cancer [8]. Although chromosomal rearrangements can be detected by next-generation sequencing of genomic DNA [9,10], RNA-Seq is a powerful tool to identify those rearrangements that lead to chimeric transcripts and are more likely to have functional consequences in cancer [3,11].

Despite these advantages of RNA-Seq, the complexity of the transcriptome and the wide dynamic range of expression levels render whole-transcriptome sequencing an expensive proposition, particularly at the depth required to call mutations and identify structural rearrangements or aberrant splice forms in low-abundance mRNAs. Mortazavi and colleagues [12] reported that 40 million reads were required to provide onefold coverage of a transcriptome, whereas the calling genotypes with high confidence may require coverage levels of at least fivefold to 20-fold [13]. This magnitude of coverage invariably results in vast oversampling of abundant transcripts, which adversely affects the efficiency and overall power of the approach.

Cost and efficiency considerations have prompted the emergence of methods that allow "targeted" next-generation sequencing. Two suitably high-throughput approaches to enrich specific sequences from genomic DNA have been developed: multiplexed molecular inversion probes (MIPs) [14-16] and capture by hybridization to oligonucleotide probes on microarrays [17-19] or in solution [20]. MIPs are similar to PCR primers in that they enrich loci defined by two flanking specific sequences. Thus, they are not appropriate for the discovery of novel chromosomal rearrangements such as translocations. By contrast, capture by hybridization can enrich DNA fragments that extend beyond the probe sequence, including sequences that are not contiguous in the reference sequence. Solution hybrid selection is a capture method that uses a complex mixture of RNA baits derived from PCR-amplified oligodeoxynucleotides to select hybridizing sequences in a library of DNA fragments [20]. To date, however, hybridization-based capture approaches have been applied primarily to genomic DNA, typically for the purpose of enriching exonic DNA of interest. Although targeted sequencing of genomic DNA facilitates mutation-discovery/profiling, it is unable to interrogate the myriad additional genomic alterations affecting DNA and mRNA that are critical to tumor biology and therapeutic development.

In this study, we explore the feasibility and power of "targeted RNA-Seq," the application of hybridization capture methods to transcriptome analysis. When applied to 467 cancer-related genes, this novel approach increased the coverage of low-abundance transcripts to levels that enabled reliable identification of sequence changes. In addition, this method provided information about relative expression levels, facilitated the discovery of novel splice variants, and enabled detection of novel fusion transcripts and isoforms thereof that would otherwise have escaped detection. As such, this method fills an important niche in cancer research, as well as other areas of genomics, by generating all the multifaceted genomic and gene-expression information in a single, straightforward experiment.

## Results

### cDNA hybrid selection

To develop a targeted RNA-Seq method, we created a complex pool of biotinylated oligonucleotide probes (baits) for cancer-related transcripts and used them to capture cDNAs from a library prepared for Illumina sequencing. We targeted 467 genes in total (887 distinct transcripts; Table S1 in Additional data file 1), representing the majority of all protein tyrosine kinase genes, nuclear hormone-receptor genes, and genes catalogued in the Cancer Gene Census [21]. Baits were designed in a tiling fashion with minimal overlap to span the entire protein-coding region of each transcript. To test the method, a cDNA library for Illumina sequencing was constructed from the K-562 chronic myeloid leukemia (CML) cell line. From an aliquot of this library, we selected cDNAs hybridizing with these cancer cDNA baits. We used PCR to regenerate a double-stranded DNA library that was sequenced in a single lane on the Illumina Genome Analyzer platform. To obtain a baseline for comparison, we also sequenced the original unenriched cDNA library.

### Sequence enrichment

**Figure 1:**
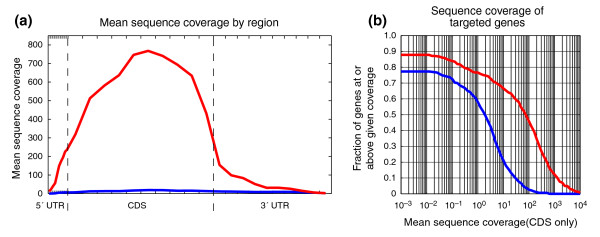
Increase in sequence coverage. **(a) **Mean sequence coverage by region. Transcript regions (5' UTR, CDS, 3' UTR) were divided into deciles, and the sequence coverage for each decile was averaged across all 887 target transcripts. Coverage is displayed for before hybrid selection (blue) and after hybrid selection (red). The average length of each region is 292, 2,136, and 1,729, respectively. **(b) **Distribution of sequence coverage for targeted genes. For each sequence-coverage threshold (x-axis), the fraction of 467 genes at or above that threshold is plotted (y-axis) for before hybrid selection (blue) and after hybrid selection (red).

Sequence analysis of the cDNA library after hybrid selection demonstrates that nearly all the high-quality, aligning reads derive from targeted genes. Approximately eight million purity-filtered [13] 76-mer sequence reads were generated for each cDNA library (before and after hybrid selection; Table 1). Reads were aligned to all curated RefSeq transcripts, requiring a unique genomic locus of origin for each placement (see Materials and methods). Hybrid selection resulted in a huge increase in specificity, with 98% of aligned reads mapping to a target transcript, versus 5% before hybrid selection (Table 1). As expected, the overall improvement in mean sequence coverage of the target transcripts was greatest for the protein-coding regions, increasing from 14.4× before hybrid selection to 606.3× after hybrid selection--a 42-fold difference (Figure 1a). The distribution in sequence coverage for the 467 target genes is shown in Figure 1b. For instance, only 62 (13%) genes achieve 20× sequence coverage before hybrid selection, whereas an additional 234 genes for a total of 296 (63%) genes are covered by at least 20× after hybrid selection. Also of note, the number of genes detectable by at least one read increases from 360 to 410 (77% to 88%). The remaining 12% of genes are probably expressed at a very low level or not at all in K-562.

**Table 1:** Analysis of Illumina sequence in cDNA hybrid selection

Sequence filter criteria	Before^a^	After^a^
Total purity-filtered reads	7,907,124	7,635,761
Aligned to all transcriptome	4,515,009	6,664,152
Unique in transcriptome	4,303,769	6,508,099
Mapping to 1 of 887 target transcripts	220,151	6,364,131
On-target specificity	5%	98%

^a^Number of sequence reads before and after hybrid selection.

This increase in sequence coverage also increased the sensitivity for detecting sequence variants in these target genes. At positions with sufficient sequence coverage, we identified nonreference bases, including SNPs and candidate mutations. Hybrid selection enabled us to identify 257 known SNPs at high confidence (LOD > 5) in the coding sequences of target genes, compared with only 76 before hybrid selection. Similarly, we identified four novel variants before hybrid selection and an additional 12 for a total of 16 after hybrid selection (Table S2 in Additional data file 2). Thirteen (81%) of the 16 were successfully validated by traditional Sanger sequencing of PCR products amplified from K-562 genomic DNA. By comparison, three (75%) of the four novel variants detected before hybrid selection were validated.

**Figure 2:**
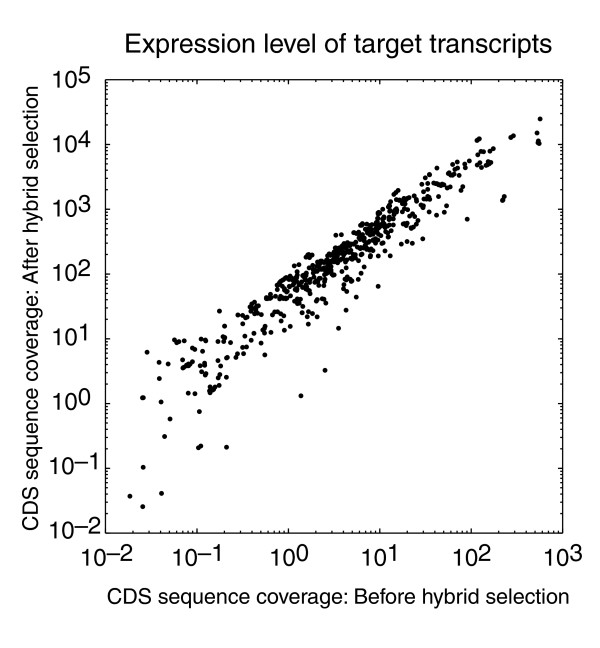
Preservation of expression levels of target transcripts in hybrid selection. For each target transcript, the sequence coverage of the coding region is plotted before and after hybrid selection.

We next asked whether the degree of enrichment for a target gene depended on its transcript abundance before hybrid selection. As shown in Figure 2, the sequence coverage observed after hybrid selection is well correlated with the sequence coverage observed before hybrid selection, indicating that the relative abundance of cDNAs from targeted genes was generally preserved. This result suggests that some expression-profiling results can be obtained simultaneously with information about sequence variants for genes targeted by hybrid selection. The correlation (*r*^2 ^= 0.71) is somewhat lower than typically observed between technical replicates of an RNA-Seq experiment [12], but comparable to the correlation between different expression profiling methods (for example, RNA-Seq and microarray hybridization) [22]. This correlation improves if the analysis is limited to transcripts in a narrower range of GC content: *r*^2 ^= 0.78 for GC 0.4 to 0.6 (645 transcripts) and *r*^2 ^= 0.87 for GC 0.45 to 0.55 (317 transcripts), indicating some bias introduced by the hybrid selection or the additional round of PCR or both.

The overall enrichment in sequence coverage for the target transcripts also enabled the identification of a greater number of alternatively spliced isoforms of these genes. Considering all possible logical intragenic combinations of exons annotated in RefSeq, 70,344 hypothetical splice junctions exist for the 467 target genes, and 6,593 of these have been annotated in RefSeq. The number of confirmed exon junctions involving target genes increased from 2,958 before hybrid selection to 4,720 after hybrid selection (Table S3 in Additional data file 3). Of these confirmed junctions, 294 are previously unannotated in RefSeq, involving 130 target genes. Genes exhibiting alternative splicing in K-562 were identified as described in Materials and methods. Hybrid selection revealed at least 177 target genes to be alternatively spliced, compared with 52 target genes before hybrid selection. Taken together, these results demonstrate the power of targeted RNA-Seq to illuminate both SNPs and splicing variants in an efficient manner.

### Fusion-transcript detection

**Figure 3:**
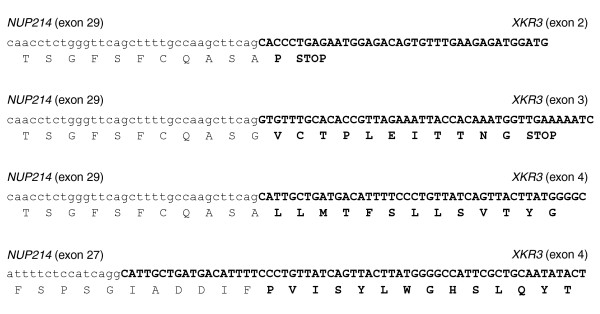
Sequences from NUP214-XKR3 fusion transcripts detected after hybrid selection. After hybrid selection, 152 reads were aligned to the transcriptome and detected as *NUP214-XKR3 *fusions. From top to bottom, we observed 137, four, eight, and three reads for these transcripts. The *NUP214 *(exon 27) to *XKR3 *(exon 4) has a stop codon downstream (not shown). Only *NUP214 *(exon 29) to *XKR3 *(exon 4) retains an open reading frame downstream of the fusion. Before hybrid selection, eight reads were aligned to the transcriptome and detected as *NUP214-XKR3 *fusions; only the *NUP214 *(exon 29) to *XKR3 *(exon 2) transcript was detected. Sequence from *NUP214 *DNA is shown as lower case, and from *XKR3*, as bold and upper case.

Because chromosomal rearrangements have important roles in cancer [8], we sought to determine whether cDNA hybrid selection could provide enhanced evidence for this class of mutations. Although K-562 has been the subject of numerous studies, until recently only the *BCR-ABL1 *translocation, which is extensively amplified [23], has been identified at the nucleotide level. We searched our cDNA Illumina data for evidence of gene fusions, or fusion transcripts composed of portions of two distinct genes (see Materials and methods). In brief, we nominated candidate fusions from 76-mer reads for which the first 30 bases and the last 30 bases uniquely aligned to separate genes, and then we searched all the reads again for 76-mers that were entirely consistent with a fusion between these two genes (requiring at least 12 bases overlap with each gene). We detected two gene fusions in the cDNA library before hybrid selection: *BCR-ABL1 *(13 reads) and *NUP214-XKR3 *(9 reads). Both gene fusions were found at similar frequencies in K-562 in a recently published RNA-Seq study [11]. After hybrid selection, *BCR-ABL1 *was implicated by 874 reads, and *NUP214-XKR3 *was implicated by 152 reads (Table 2 and Figure 3). Although *NUP214 *fusions have been observed previously in tumors and other cell lines [11,24,25], *NUP214-XKR3 *is of particular interest because it shows that we can enrich for fusion transcripts for which only one of the genes, *NUP214*, was directly targeted by the hybrid-selection baits. The *NUP214-XKR3 *reads derive from one end of a larger fragment, and the orientation of the reads indicates that the cDNA fragments were composed mostly of *NUP214 *sequence (Table S4 in Additional data file 4). This bias in sequence composition of fusion-transcript cDNA fragments is as expected because the baits target only the *NUP214 *sequence. Another important finding is that this method enabled detection of three additional *NUP214-XKR3 *fusion-transcript isoforms in 7.6 million reads (Figure 3 and Table 1) that were not detected without hybrid selection nor in the 20.7 million reads in the recent K-562 RNA-Seq study [11]. All four of the *NUP214-XKR3 *fusion transcripts were confirmed by Sanger sequencing RT-PCR products (data not shown). It is interesting to note that only one of the four *NUP214-XKR3 *fusions maintains an open reading frame downstream of the fusion event (Figure 3). This fusion was not detected by sequencing the cDNA library before hybrid selection nor in the recent K-562 RNA-Seq study [11]. Understanding the functional significance of these fusion transcripts is beyond the scope of this study, but this work clearly demonstrates the power of targeted RNA-Seq to detect them.

**Table 2:** Hybrid selection-enhanced detection of fusion transcripts

5' Gene	**5' Chr**.	3' Gene	**3' Chr**.	Before^a^	After^a^
*BCR*	22	*ABL1*	9	13	874
*NUP214*^b^	9	*XKR3*^b, c^	22	9	152
*SNHG3-RCC1*^b, c^	1	*PICALM*^b^	11	1	39
*PRIM1*^c, d^	12	*NACA*^b, d^	12	0	22
*NCKIPSD*^ d^	3	*CELSR3*^c, d^	3	0	5
*SLC29A1*^c, d^	6	*HSP90AB1*^ d^	6	0	2

^a^Number of sequence reads before and after hybrid selection. ^b^Fusion transcript reads identified from more than one exon in this gene. ^c^Not included in hybrid selection bait genes. ^d^Not previously annotated, read-through transcripts between adjacent genes.

In addition to *BCR-ABL1 *and *NUP214-XKR3*, which were both detected before hybrid selection, we identified four gene fusions after hybrid selection that may have otherwise gone undetected and were not found previously [11]. In each case, only one of the two genes was specifically targeted by baits (Table 2 and Table S4 in Additional data file 4). Three of the four gene fusions involve the production of "read-through" transcripts in which exons from separate, adjacent genes are joined together in a single mRNA molecule. Read-through transcripts have previously been discovered in cancer and have been shown to contribute to tumorigenicity [3]. The fourth novel gene fusion involves the previously annotated *SNHG3-RCC1 *read-through transcript on chromosome 1 and *PICALM *on chromosome 11. As with *NUP214-XKR3*, multiple splice isoforms were detected for *SNHG3-RCC1*-*PICALM*, and four of five of them were confirmed by sequencing RT-PCR products (data not shown). Although these observations are consistent with a single genomic translocation followed by alternative splicing of the resulting RNA in both cases, it also is possible that further amplifications and rearrangements at this locus contributed to the multiple fusion transcripts observed.

## Discussion

In this study, we demonstrated that combining hybridization capture of a cDNA library with Illumina sequencing provides a robust and sensitive method to detect a wide range of DNA and RNA sequence alterations present in cancer cells. First, this method has a very high specificity, as 98% of the sequence mapping to RefSeq aligns to targeted transcripts after hybrid selection (Table 1). Second, this selectivity leads to improved detection of SNPs (Figure 1), splice isoforms, and fusion transcripts (Table 2) in the targeted transcripts. Importantly, this property reduces the amount of sequencing, and consequently costs, required to identify mutations and other cancer-associated variants. Third, differences in transcript abundance are generally preserved after hybrid selection (Figure 2), likely because the baits are in molar excess during the hybridization [20]. Similarly, preservation of genomic copy-number alterations (that is, amplifications and deletions) has been observed after hybrid selection of genomic DNA in cell lines with well-characterized chromosomal aberrations (M.F.B. and L.A.G., unpublished data) and in filter-based hybridization experiments [26]. Fourth, information that reflects function, such as expression levels, alternative splicing, and RNA editing, can be obtained by RNA-Seq directly from RNA input material rather than from genomic DNA. Beyond RNA expression levels (Figure 2), it is also possible to demonstrate that a particular fusion transcript is expressed, to identify fusion transcripts with partner genes that are not in targeted baits, and even to show the relative abundance of different spliced fusion transcripts (Table 2 and Figure 3). Fifth, fusion transcripts due to trans-splicing [27] would also be detected by this method, but not by genomic sequencing, and could be distinguished from translocations by validation experiments with genomic DNA. In summary, by sequencing cDNA rather than genomic DNA, we generated a richer view of the biologic state of this cell line. Recent studies that used MIPs to select for sequences subject to RNA editing [28] or to analyze allele-specific expression [29] provide additional examples of how targeted RNA-Seq can enhance our understanding of the molecular state of the transcriptome. Finally, this method is easily scalable to larger numbers of samples and genes.

Our targeted RNA-Seq method provides a direct and powerful approach to discover and characterize translocations and to study their prevalence in all types of cancer [8], including solid tumors, which is an area of active research [3,4]. Although the role of translocations in leukemias and sarcomas is well established, we were able to identify novel fusion transcripts in the well-studied K-562 CML cell line (Table 2). By enriching for cDNA sequences from genes of known relevance to cancer, targeted RNA-Seq makes possible the identification of translocations for any number of targeted genes in a single experiment. In addition, oncogenes often have multiple translocation partners [8], and this method provides an effective tool to identify new partners for genes previously identified in translocations, because only one of the two translocated genes must be present in the hybrid-selection baits. This method is able to recover fusion transcripts in which incomplete matches to baits exist, probably because baits adjacent to the bait whose sequence contains the breakpoint enable this recovery and enrichment. This may be possible because the cDNA library inserts are 290 to 390 bp, which is larger than the 170-base baits.

It is interesting to compare the efficacy of targeted RNA-Seq to enhance detection of low-abundance transcripts with that of cDNA library normalization. Normalization is better suited for discovery of sequence changes in transcripts not known to be associated with a particular biologic question. By contrast, targeted RNA-Seq is ideal for increasing coverage for a subset of "high interest" transcripts. Further, unlike normalization, targeted RNA-Seq preserves expression-level information (Figure 2). In addition, targeted RNA-Seq can achieve higher increases in coverage for a subset of targeted transcripts, depending on the number of unique baits designed. If coverage of lower abundance transcripts is a priority in a given experiment, information about transcript abundance can be used during bait design to focus on those transcripts with targeted RNA-Seq. Conventional normalization methods [30,31] are unlikely to achieve easily the approximately 30-fold enrichment for most low-abundance transcripts observed in our targeted RNA-Seq experiments (Figure 1; JZL., XA, unpublished results).

## Conclusions

By combining hybridization capture of cDNAs and next-generation sequencing, targeted RNA-Seq provides an efficient and cost-effective means to analyze a specific subset of a transcriptome simultaneously for mutations, structural alterations, and expression levels. This method overcomes the limitations of conventional RNA-Seq that requires significantly greater sequencing depth to generate sufficient coverage of low-abundance transcripts. It also circumvents certain limitations of targeted genomic DNA sequencing, in which detection of chromosomal rearrangements may be challenging (and analysis of mRNA effects is impossible). In a single experiment, targeted RNA-Seq provides a wealth of qualitative as well as quantitative information that cannot be obtained by any single other method. Targeted RNA-Seq is therefore a powerful and convenient new approach that is well suited for a wide range of large-scale tumor-profiling studies in many clinical or research settings.

## Materials and methods

### Illumina library construction and sequencing

A K-562 cDNA library (insert size of 290 to 390 bp) for Illumina sequencing was constructed from a 500 ng aliquot of double-stranded cDNA prepared from 3 μg polyA^+ ^RNA (Ambion, Austin, TX USA) primed with 0.3 μg random hexamers (Invitrogen, Carlsbad, CA USA), as described previously [22], except (a) no RNase inhibitor was used, (b) low-intensity shearing was performed for 5 seconds rather than 4 seconds, and (c) PCR primers were removed with 1.8× volumes of AMPure beads (Agencourt Bioscience Corporation, Beverly, MA USA). We used 14 PCR cycles to generate the library before hybrid selection and an additional 18 cycles afterward with the same PCR conditions. Single reads of 76 bases were generated on an Illumina Genome Analyzer II. The raw unaligned Illumina sequences in SRF (sequence-read format) are available at [32].

### Bait design and synthesis

We designed 11,566 bait sequences (Table S5 in Additional data file 5) targeting the coding sequence of 887 transcripts of 467 genes described in the NCBI RefSeq database. The RefSeq file used contained 45,376 transcript sequences from all NM and XM human transcripts (downloaded from [33] on June 23, 2008). Each bait was composed of 170 bases matching the transcript sequence it was intended to enrich. Baits were tiled across the coding region of each transcript, from 5' to 3', such that all coding bases were covered by at least one bait and that overlap between baits was minimized and distributed evenly among all baits. The median and mean overlaps between adjacent baits were five and seven bases, respectively. Where genes existed with multiple splice variants, baits were designed for each splice variant independently, so that 9,913 unique baits were designed. The baits were synthesized by Agilent Technologies (Santa Clara, CA USA) on a custom 55,000 spot array. To fully use the array, the 11,566 baits were replicated so that at least two copies of each oligonucleotide were ordered, plus two copies of the reverse complement of each oligonucleotide. Oligonucleotides and their reverse complements give rise to the same PCR products. Thus, although sense and antisense oligonucleotides were chemically synthesized, only sense RNA baits were present in the hybridization.

### Hybrid selection

Five hundred nanograms of the K-562 cDNA Illumina library was selected as described previously [20], except that the MEGAshortscript T7 Kit (Ambion) was used for the bait preparation.

### Sequence alignment and coverage

Purity-filtered [13] 76-mer reads were aligned to all curated protein-coding transcripts in RefSeq (downloaded from [33] on March 1, 2009) allowing up to four mismatches, and mapped back to their genomic coordinates in the reference human genome (hg18), preserving splice junctions. Alignments were performed by using the ImperfectLookupTable (ILT) tool of the ARACHNE genome assembly suite [34]. Reads were considered informative if all placements to RefSeq transcripts originated from a unique genomic locus, and the next-best placement contained at least three additional mismatches. Sequence coverage was determined separately for 5' UTRs, coding sequences (CDS), and 3' UTRs.

### Sequence variant identification

To eliminate false positives in calling mutations, reads aligning to RefSeq transcripts were also aligned directly to the genome, and uniqueness was required in both the transcriptome and the genome. Each position was assigned a LOD score indicating the likely accuracy of the call, according to the observed sequence coverage, allele distribution, and reference base [20]. Of 1,085,748 bases in the coding sequence of targeted genes, 297,693 bases exhibited LOD greater than 5 before hybrid selection, and 724,211 bases exhibited LOD greater than 5 after hybrid selection. Bases that disagreed with the reference genome were classified as known SNPs if present in dbSNP [35] (build 129) or as novel variants. Novel variants were discarded if they occurred within five bases of another novel variant (to compensate for alignment artifacts produced by indels), if they were observed on Illumina reads in only one orientation, or if they fell within segmental duplications [36]. The remaining novel variants were considered high confidence and submitted for validation (see later).

### Splice isoform identification

To catalog the exon junctions detected by RNA-Seq, we created a database of all hypothetical intragenic exon junctions involving RefSeq genes. Each 76-mer read was aligned to this new reference-sequence database in the same manner as described earlier. Exon junctions were "confirmed" in K-562 if they harbored at least two distinct 76-mer reads mapping to the junction with, at most, four mismatches but with at least 10 mismatches with their best placement on the genome. Those genes with at least two confirmed exon junctions that overlapped each other (for example, one upstream exon joined to two downstream exons, two upstream exons joined to one downstream exon, or alternating exons) were considered to be alternatively spliced.

### Fusion transcript identification

To identify candidate gene fusions from individual 76-mer reads, the first 30 and last 30 bases were separately aligned to all curated protein-coding transcripts in RefSeq (allowing up to two mismatches). Reads for which both ends mapped to separate genes were flagged for further analysis. Gene pairs implicated by at least two distinct reads (for which the orientation was consistent with a gene fusion) were nominated as candidate fusions. The entire set of 76-mer reads was then searched for instances joining any exon of the upstream gene to any exon of the downstream gene across the full 76 bases, requiring at least 12 bases overlap with each gene. To call confidently a gene pair as a fusion event, we required at least two distinct instances that could not be placed anywhere else in the transcriptome or the genome. The criteria used are conservative to avoid false-positive fusion transcript alignments; additional fusion transcripts may be present, but not detectable with these alignment parameters and coverage levels.

### Validation of sequence alterations

Novel SNPs called by RNA-Seq were validated by traditional bidirectional Sanger sequencing of PCR products that had been amplified from 20 ng of K-562 genomic DNA by 35 PCR cycles with Herculase Hotstart DNA polymerase (Stratagene, La Jolla, CA, USA).

For *NUP214-XKR3 *and *SNHG3-RCC1*-*PICALM *fusion transcripts, confirmation was attempted by Sanger sequencing of RT-PCR products. First-strand cDNA was synthesized from K-562 mRNA with random hexamers (Invitrogen) or gene-specific primers (Eurofins MWG Operon, Huntsville, AL, USA) as described earlier. For random priming, 1 μg mRNA and 1.5 μg random hexamers were used, and for gene-specific priming, 500 ng mRNA and 2 pmol gene-specific primers were used. The cDNA was purified by using 1.8× volume of Agencourt AMPure PCR Purification kit. Fusion transcript-containing cDNAs were then amplified by 30 to 40 PCR cycles by using 1/50 of the purified first-strand cDNA, 25 pmol forward and reverse gene-specific primers, 100 μmol Betaine (Sigma-Aldrich, St. Louis, MO USA), and Phusion Master Mix with GC Buffer (New England BioLabs, Ipswich, MA, USA) in a 50 μl volume. PCR products were gel purified from a 10% TBE Criterion Gel (BioRad, Hercules, CA, USA). Gel slices were excised, crushed, and eluted with 250 μl 0.3 *M *NaCl for more than 4 hours followed by ethanol precipitation. The purified PCR products were sequenced as described earlier and compared with junctions identified by Illumina sequencing. All primer sequences are available on request.

## Abbreviations

CDS: coding sequence; CML: chronic myeloid leukemia; ILT: ImperfectLookupTable; MIP: molecular inversion probe; SRF: sequence read format.

## Authors' contributions

JZL and MFB wrote the article. XA, AM, AG, CN, and LAG assisted in editing the article. MFB chose the targeted genes, and TF designed the baits. XA prepared the Illumina cDNA libraries. PR and AM performed the hybrid selection. MFB and JZL analyzed the sequence data. XA and AG confirmed fusion transcripts and SNPs, respectively. JZL, CN, LAG, and AG conceived and directed the research.

## Additional data files

The following additional data are available with the online version of this article: a table listing the bait gene names and transcript accession numbers (Additional data file 1), a table listing novel SNPs (Additional data file 2), a table listing splice junctions (Additional data file 3), a table listing Illumina reads from fusion transcripts (Additional data file 4), and a table listing the bait sequences (Additional data file 5).

## Supplementary Material

Additional data file 1 Bait gene names and transcript accession numbers Click here for file

Additional data file 2 Novel SNPs Click here for file

Additional data file 3 Splice junctions Click here for file

Additional data file 4 Illumina reads from fusion transcripts Click here for file

Additional data file 5 Bait sequences Click here for file
